# Diet in Cholera Times

**Published:** 1855-08

**Authors:** 


### DIET IN CHOLERA TIMES.

If you have no special liking for one thing more than another, and have not even the premonitory symptom, to wit, the *belly-uneasiness*, then the following diet will render you more secure:

Breakfast.—A single cup of weak coffee or tea, with toasted bread, or cold bread and butter, and a small piece of salt meat, ham, beef, fish, or the like, *and nothing else.* Dinner—Cold bread, roasted or broiled fresh meat of some kind, potatoes, rice, hominy, samp, or thickened gruel. For Dessert—Rice, or bread pudding, or sago, arrow root, tapioca, farina, corn starch, prepared in the usual manner, *and nothing else fluid or solid.* Tea, or Supper—A single cup of weak tea of some kind, or coffee, with cold bread and butter—*nothing else.*

Eat nothing between meals; go to bed at a regular early hour, not later than ten o'clock; attend to your business with great moderation, avoiding hurry, bustle, worriment of mind; wear thin woollen flannel next the body during the day, air it well at night, sleeping in a common cotton night garment; remain in bed of mornings, after you have waked up, until you feel rested in all your limbs; but do not by any means take a second nap. Do not sleep a moment in the day time, and let all your enjoyments and recreations be in great moderation.

Fruits have not been named, because it is so difficult to get them fresh, ripe, perfect—many looking so, are wormy. Except potatoes, no vegetables are named, because they more readily sour on the stomach, require more power of digestion, while they do not afford as much nutriment and strength to the body in proportion.

